# Antimicrobial Performance of a Novel Drug-Eluting Bioenvelope

**DOI:** 10.3390/antibiotics14040330

**Published:** 2025-03-21

**Authors:** Zerelda Esquer Garrigos, Sunil Kapur, Michelle LeRoux Williams, M. Rizwan Sohail

**Affiliations:** 1 Division of Infectious Diseases, Department of Medicine, University of Mississippi Medical Center, Jackson, MS 39216, USA; 2 Division of Infectious Diseases, Department of Medicine, Mayo Clinic College of Medicine and Science, Rochester, MN 55905, USA; 3 Cardiac Arrhythmia Service, Division of Cardiovascular Medicine, Brigham and Women’s Hospital, Harvard Medical School, Boston, MA 02115, USA; 4 Elutia Inc., Silver Spring, MD 20904, USA; 5 Section of Infectious Diseases, Department of Medicine, Baylor College of Medicine, Houston, TX 77030, USA

**Keywords:** antibacterial envelope, cardiac implantable device envelope (CIED), neurostimulator, drug delivery, biologic envelope, AATCC

## Abstract

**Background:** Preventing infections associated with cardiac implantable electronic devices (CIED) and neurostimulators is essential to optimizing patient outcomes. This study aimed to evaluate the antimicrobial performance of a biologic CIED envelope incorporating a bioabsorbable disc infused with rifampin and minocycline. **Methods**: The antimicrobial activity was evaluated in a rabbit model and in vitro elution tests. Based on in vivo–in vitro correlation studies, a modified AATCC-100 method was used to quantitatively assess antibacterial activity across seven bacterial strains relevant to CIED infections. **Results**: Pharmacokinetic analysis showed a biphasic elution profile, with rapid initial release followed by more gradual elution over 14 days. The AATCC results showed no bacterial recovery for any tested species, with complete eradication in all replicates. **Conclusions**: These results support the use of antibiotic-eluting bioenvelopes as an effective strategy for preventing bacterial infections associated with CIED. The modified AATCC-100 test and in vivo–in vitro correlation studies provide new tools for the evaluation of the antibiotic activity of implantable biomaterials.

## 1. Introduction

Infections associated with medical devices, such as cardiac implantable electronic devices (CIEDs) and neurostimulators, present significant risks to patients, leading to complications and increased healthcare costs [1,2,3]. Preventing device-related infections is crucial in reducing the frequency of healthcare-associated infections and optimizing patient outcomes. Consequently, developing more effective methods to safeguard medical devices, particularly those intended for implantation or prolonged contact with the body, is imperative.

One approach to mitigating the risk of device-related infections is the use of antibiotic-eluting biologic matrices [4,5]. These matrices are designed to release antimicrobial agents, such as rifampin and minocycline, to prevent bacterial colonization of device surfaces and subsequent infection. Rifampin and minocycline, when administered in combination, offer broad-spectrum antimicrobial coverage; rifampin exhibits potent activity against Gram-positive pathogens, particularly *Staphylococcus* species, while minocycline extends coverage to Gram-negative organisms [6,7,8,9]. This synergistic combination leverages the distinct mechanisms of action of each drug, thereby enhancing antimicrobial efficacy and reducing the risk of developing antimicrobial resistance.

A next-generation product has been developed that combines antibiotic elution with the regenerative capabilities of extracellular matrix (ECM) biomaterials to create an antibiotic-eluting bioenvelope. This innovative drug delivery system was designed to minimize any adverse physical or chemical effects of antibiotic impregnation on the ECM. Instead of using a conventional drug coating or impregnation method, a drug-eluting disc composed of a bioabsorbable polymer was integrated into the biologic envelope. This straightforward and effective solution ensures that key ECM properties, such as surface characteristics and porosity, remain unaltered, thereby supporting cell infiltration, angiogenesis, and tissue integration [10,11,12].

To study the antibacterial performance of this novel bioenvelope, the AATCC Test Method 100, established by the American Association of Textile Chemists and Colorists (AATCC), was utilized [13]. Originally designed to assess the antibacterial properties of textiles, the principles of AATCC-100 have been adapted and applied to other materials, including medical devices, such as wound dressings [14]. Application of AATCC-100 to the antibiotic bioenvelope is appropriate due to the fabric-like texture of the bioenvelope’s intact ECM.

The AATCC-100 method is a quantitative method for assessing antibiotic efficacy that involves exponential-phase cell growth of bacteria, inoculation onto wound dressing samples, and neutralization of the biocidal agent [13]. Cell concentration is determined by counting colony-forming units (CFUs) and calculating log reduction normalized to control material and/or the starting inoculum. The two recommended organisms for AATCC-100 testing are *Pseudomonas aeruginosa* and *Staphylococcus aureus*, pathogens commonly found in wound infections [14]. *S. aureus*, a Gram-positive bacterium, is a leading cause of surgical site infections and pneumonia and a major cause of bloodstream infections [15,16,17]. For this study, the test has been extended to include additional organisms commonly identified in CIED-related infections. An additional objective is to demonstrate the broad-spectrum efficacy of the antibiotic bioenvelope, including against Gram-negative bacteria, which can be particularly difficult to eradicate.

In this study, the AATCC-100 method was adapted to simulate in vivo conditions beyond the 1-week endpoint often used in animal models. By preconditioning the antibiotic bioenvelope to simulate conditions after 2 weeks in vivo, this novel testing method aims to provide a more rigorous assessment of the device’s antibacterial activity under physiologic conditions. This approach addresses a critical challenge: evaluating the device’s performance even after much of the antibiotic has been eluted as the disc undergoes biodegradation. An in vivo–in vitro correlation ensures the relevance of the test results to real-world conditions, offering several advantages over traditional testing methods, including standardization, real-world relevance, and quantitative evaluation that does not rely on the diffusion properties observed in methodologies such as time-kill testing. This approach aims to enhance our understanding of how the device performs clinically by integrating in vitro findings with observed in vivo preclinical data.

The purpose of this study is to demonstrate the robustness of the antibacterial activity of a drug-eluting biomatrix, which has significant implications for preventing infections associated with subcutaneous device implants. The results of this study provide insights into the antibacterial properties of the antibiotic bioenvelope, contributing to the overall understanding of its performance and safety profile. Additionally, the study tracks changes in antimicrobial efficacy over time to understand the effects of device aging and provide assurance of performance over extended time periods. Finally, this research may encourage the adoption of standardized testing methods, such as AATCC-100, in the evaluation of other medical devices, facilitating comparability and ensuring quality across different products.

## 2. Results

### 2.1. In Vivo Animal Pharmacokinetics Study

**Figure 1:**
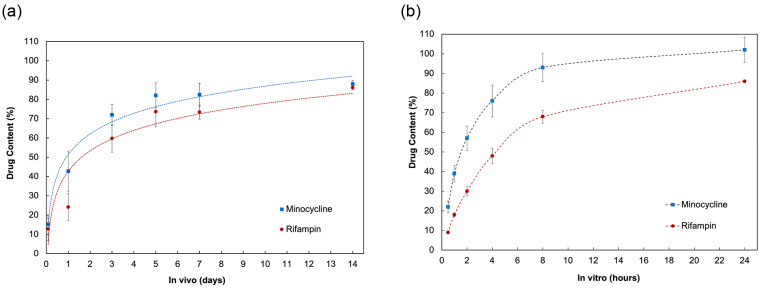
In vivo and in vitro drug elution used to develop in vivo–in vitro correlation and determination of preconditioning parameters. (**a**) The in vivo data were generated from a rabbit subcutaneous implant model, in which residual drug content in the device was measured at predefined time points. Data are presented as mean ± standard deviation (*n* = 4). (**b**) The in vitro procedure involved drug elution under physiologic conditions. Data are presented as mean ± standard deviation (*n* = 3).

In the rabbit subcutaneous dorsal implant model, the antibiotic bioenvelope effectively isolated and stabilized the dummy CIED. Pharmacokinetic analysis revealed continued elution of rifampin and minocycline throughout the 14-day period post-implantation (Figure 1). Measurement of residual drug content in explanted devices at various time points demonstrated the extent of drug retention and release kinetics. Initially, there was a steep incline in the release profile, indicating an increasing rate of drug elution. Median drug release was achieved by day 2 for both rifampin and minocycline. Drug release continued over the 2-week period at a slower rate. The cumulative release at 2 weeks was similar for both antibiotics, accounting for 86% of total rifampin and 88% of total minocycline.

### 2.2. In Vitro Elution of Antibiotics

**Figure 2:**
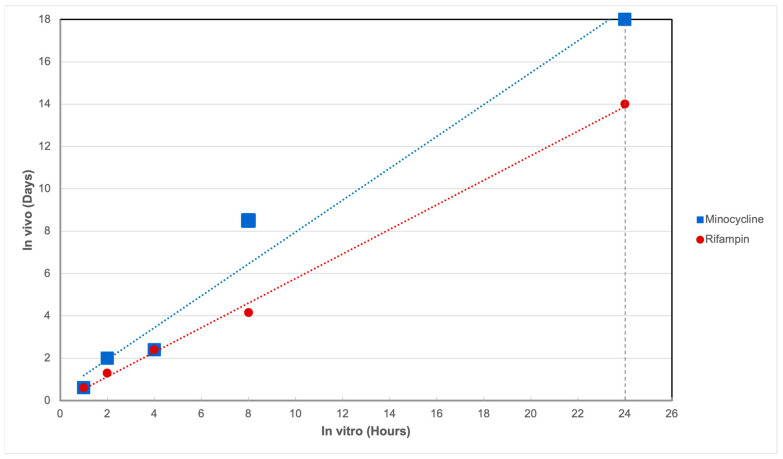
In vivo–in vitro correlation demonstrating the rationale for a 24 h preconditioning period, corresponding to a minimum of 2 weeks post-implantation in vivo. The preconditioning process ensures controlled elution of rifampin and minocycline, aligning the antibiotic levels with the time of interest in vivo. Strong linear correlations were observed between in vitro and in vivo elution times, with R^2^ values of 0.998 for rifampin and 0.972 for minocycline. Linear correlation was assessed using five data points for each drug. Dotted lines represent regression lines for rifampin and minocycline. Note: Some data points overlap due to similar elution values at certain timepoints, reducing the number of visually distinct points.

In vitro elution studies demonstrated controlled and sustained release of rifampin and minocycline from the antibiotic bioenvelope. The shape of the in vitro elution profile was similar to the in vivo profile, characterized by an initial logarithmic release followed by slowing over time (Figure 2). Minocycline exhibited a faster release rate, particularly in vitro, attributable to its higher solubility in physiologic media.

The in vitro elution samples showed a gradual increase in antibiotic concentration, reaching peak levels at 24 h. Median elution was achieved at 4 h. Over time, the rate of drug release slowed and cumulative drug release plateaued, with minocycline achieving 102% elution and rifampin 86% elution by the final time point of 24 h.

The binding of rifampin and minocycline to the matrix at equilibrium was assessed by analyzing the drug content of the bioenvelope after 7 days at 37 °C under physiologic conditions. Drug assay analysis identified a mean minocycline content in the disc of 6.5% and a mean rifampin content of 13.8% after the 7-day period (Table 1). On average, 9.6% of minocycline and 11.8% of rifampin were bound to the matrix, highlighting the bioenvelope’s ability to retain meaningful levels of these drugs in the ECM.

### 2.3. In Vitro–In Vivo Correlation

The IVIVC study demonstrated a strong relationship between the levels of drug elution in vitro and the measured residual drug in vivo. The Pearson correlation coefficient was 0.999 for rifampin and 0.986 for minocycline, indicating a high degree of linear correlation between the in vitro elution data and drug levels measured in the rabbit model. By plotting the in vivo–in vitro relationship in a manner analogous to a Levy plot, the extent of in vitro elution can be related to the amount of drug remaining at different times in vivo (Figure 2).

The preconditioning regimen was based on an analysis that showed the amount of drug remaining at 7 days post-implantation was equivalent to 12 h of IVE. To simulate a more rigorous scenario for AATCC testing, the preconditioning was extended to 24 h, corresponding to approximately 2 weeks post-implantation in vivo. This approach ensured that the AATCC testing demonstrated the ability of the device to reduce bacterial colonization even after an extended elution period equivalent to 2 weeks in vivo. The preconditioning process provided for the controlled elution of rifampin and minocycline until the antibiotic levels corresponded to the desired in vivo time point.

### 2.4. Modified AATCC Test Method 100 Study

The antibacterial activity of the antibiotic envelope was evaluated using the modified AATCC Test Method 100 across seven bacterial strains. Based on the bacterial growth achieved following inoculation, the absolute starting bioburden ranged from 10^5^ to 10^8^ CFU, as determined by recovery from the non-drug control bioenvelopes. Growth promotion controls confirmed the viability of all bacterial strains.

**Figure 3:**
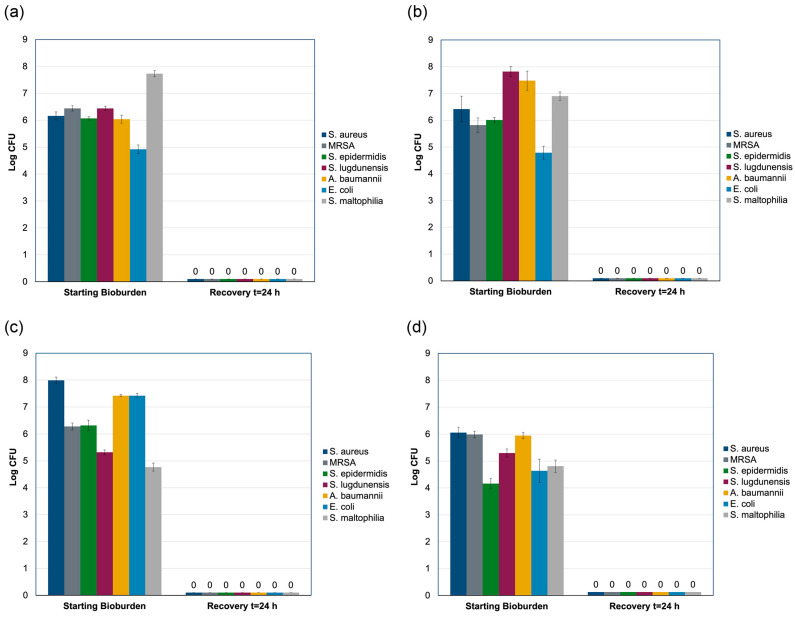
Modified AATCC 100 results for aged samples of antibiotic bioenvelope. Aging time was (**a**) 3 months, (**b**) 6 months, (**c**) 9 months, and (**d**) 12 months. No bacterial recovery was observed after 24 h culture. Each test included three replicates. The mean of the three replicates is reported and error bars represent standard deviation.

The AATCC results indicated no bacterial recovery for any of the bacterial species tested. Figure 3 illustrates the ability of antibiotic bioenvelope to prevent bacterial colonization of the biomaterial. Complete eradication was observed in each of the three replicates for each bacterial species tested. Furthermore, the device’s antibacterial activity was sustained over time, as evidenced by the real-time aging studies conducted at 25 °C. At each aging interval (3, 6, 9, and 12 months), the device exhibited complete eradication of all organisms tested, with no bacterial recovery observed at any time point.

## 3. Discussion

Infections associated with implantable devices, such as CIED and neurostimulators, are a serious complication that can result in significant morbidity and mortality and increased healthcare costs [3,18]. The highest risk of contamination occurs during implantation, emphasizing the critical need for localized delivery of antibiotics directly to the subcutaneous pocket in the immediate post-operative period. The antibiotic bioenvelope tested in this study addresses this challenge by effectively isolating and securing implantable devices while continuously releasing elevated concentrations of broad-spectrum antibiotics (minocycline and rifampin) into the pocket. This proactive approach aims to minimize infection risk and mitigate the severe consequences associated with device-related infections.

The results of this study demonstrated the robust antibacterial performance of the antibiotic bioenvelope. In the rabbit model, the bioenvelope effectively isolated and secured the implanted device while showing initial rapid antibiotic elution into the pocket, followed by continued release over a two-week period. In vitro analyses confirmed substantial antibacterial efficacy of the antibiotic bioenvelope against a spectrum of clinically relevant bacterial strains associated with CIED infections. Importantly, it consistently prevented bacterial colonization even when challenged with high bacterial loads ranging from 10^5^ to 10^7^, and it eradicated all tested organisms. This high level of antibacterial activity was maintained throughout the 12-month product aging period.

These findings highlight that complete bacterial eradication persisted after in vitro preconditioning of the antibiotic bioenvelope to simulate antibiotic levels remaining after 2 weeks of implantation in vivo. This suggests that it should remain effective in eliminating bacteria in the pocket even after the release of the majority (>80%) of antibiotics from the device. The kinetics of drug release in this preclinical challenge study appear to meet the clinical requirement for maintaining high local antibiotic levels post-implantation, characterized by an initial phase of rapid drug release followed by sustained elution. The observed durability of these effects may be aided by the binding of antibiotics to proteins within the matrix. The absence of failures and the complete eradication of all organisms in the study underscore the robust antibacterial efficacy of the antibiotic bioenvelope, suggesting their potential to significantly reduce the risk of device-related infections in clinical settings. Together, these results provide robust preclinical evidence supporting the potential of the antibiotic bioenvelope to offer protection against bacterial contamination and the associated infection risks linked with CIED implants.

The novel testing method used in this study, which bridges in vitro and in vivo conditions, provides a realistic assessment of the antibacterial activity of the antibiotic bioenvelope under physiologic conditions, even after most of the antibiotics have eluted. The correlation study established a strong relationship between in vitro and in vivo data, supporting the reliability and relevance of this testing method for predicting real-world performance of drug-eluting biomaterials in the in vitro setting.

These results support the findings of previous preclinical and clinical studies of antibiotic-eluting envelopes for infection prevention [4,7,19,20,21,22]. Preclinical studies of bioenvelopes impregnated with antibiotics have reported a similar biphasic pattern of antibiotic release, with an initial bolus followed by sustained release for several days [4,5]. As noted, this pattern should be ideal for the prevention of infection, as bacterial contamination most likely occurs at the time of implantation. In terms of clinical performance, meta-analyses report >60% reductions in major CIED infections with different types of antibiotic-eluting envelopes [19,20,21].

The local drug delivery provided by this combination drug-device construct has several advantages over systemic drug administration. By minimizing systemic exposure, this approach reduces risk for side effects and the need for frequent dosing associated with systemic antibiotics. Rifampin and minocycline are highly effective against relevant pathogens, with MIC values for *Staphylococcus aureus*, *Staphylococcus epidermidis*, and other clinically relevant organisms typically ranging from 0.008 to 1 µg/mL for rifampin and 0.12–4 µg/mL for minocycline [23,24]. Maintaining effective concentrations at the local site may also help to prevent the development of antibiotic resistance, as it reduces the likelihood of subtherapeutic exposure that can promote resistant strains.

The antibacterial efficacy and clinical utility of the antibiotic bioenvelopes are supported not only by the inclusion of antibiotic-eluting discs, but by the biomaterial itself. The bioenvelope is composed of decellularized, non-crosslinked ECM derived from porcine SIS, which has been demonstrated to create a supportive environment for tissue integration and vascular ingrowth [11,25,26]. With regard to antibiotic efficacy, the natural remodeling of intact ECM following implantation releases bioavailable growth factors and antimicrobial elements, which support tissue integration and the suppression of bacterial infection [27,28,29]. Intact ECM naturally modulates the immune response to mitigate inflammation and promote the tissue vascularization and integration essential for optimal outcomes post-implantation [30,31]. Indeed, the ECM bioenvelope was specifically designed to stabilize the device within the subcutaneous pocket through the development of healthy, vascularized tissue, which may limit risk for device migration or erosion, and possibly facilitate device removal when future exchange or revision is required [10,32]. This constructive remodeling of intact ECM contrasts with the robust foreign body response to implanted synthetic surgical materials, which leads to chronic inflammation and encapsulation of the material in fibrotic, hypovascularized tissue [3,31,33,34,35,36].

This study was limited by the use of discrete time points for measuring drug release in the animal model. While these intervals provided valuable data, more frequent or continuous monitoring, such as with microdialysis technology, could provide more detail on the release profile and more precise characterization of drug release kinetics over time. This technique could improve pharmacokinetic modeling by capturing transient fluctuations and providing a smoother, more accurate release curve, which is difficult to achieve through isolated sampling. The in vitro to in vivo correlation model successfully demonstrated the utility of bench testing for predicting in vivo behavior. However, incorporating more complex tissue models could further improve predictive accuracy. Additionally, the in vitro to in vivo correlation model may not fully capture the complexities of clinical settings, as factors such as tissue absorption and blood flow could influence the antibiotic release rates observed in vivo.

Overall, the local drug delivery system presented in this study offers a promising approach for the treatment and prevention of infections associated with implantable devices.

## 4. Materials and Methods

This study was conducted in compliance with Good Laboratory Practice (GLP) regulations and ISO-10993-6 guidelines [37]. All animal procedures were conducted in accordance with institutional guidelines and approved by the Institutional Animal Care and Use Committee (IACUC). The study adhered to the principles outlined in the Guide for the Care and Use of Laboratory Animals.

**Figure 4:**
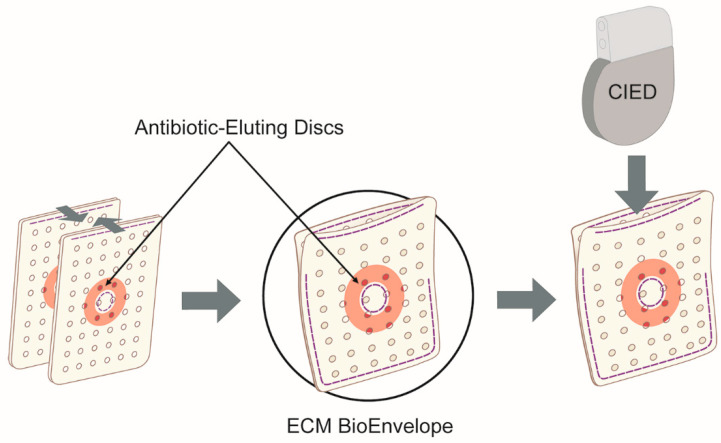
Diagram of the antibiotic-eluting bioenvelope, containing a drug-eluting, biodegradable polymer disc with rifampin and minocycline, in multilaminate ECM sheets. The polymer disc is made of PLGA. The bioenvelope is constructed from two perforated sheets and is designed for the insertion of CIEDs or neurostimulation devices.

The device under study is an antibiotic bioenvelope (8.0 cm × 6.9 cm) designed to support and stabilize CIED and other implantable devices post-surgery (Figure 4). It is constructed from perforated sheets of decellularized, non-crosslinked, lyophilized ECM derived from porcine intestinal submucosa, combined with drug-eluting discs (EluPro™, Elutia Inc.; Silver Spring, MD, USA). The drug-eluting discs are made of poly(lactide-co-glycolide) (PLGA) infused with rifampin and minocycline. PLGA is a well-described biodegradable polymer technology for controlled release of drugs in vivo [38,39]. The 25 mm discs are engineered to provide extended drug release, with a minimum nominal content of 95 µg/cm^2^ rifampin and 85 µg/cm^2^ minocycline.

### 4.1. In Vivo Pharmacokinetics Study

The New Zealand White Rabbit subcutaneous dorsal implant model was utilized for this study, as described previously [40]. This study utilized both male and female (nulliparous and non-pregnant) animals, with an initial procedure weight range of 2.0–3.0 kg. This model enables thorough evaluation of the ability of the antibiotic bioenvelope to isolate and stabilize CIED and other subcutaneous devices [40]. The anatomical similarity of the rabbit’s subcutaneous dorsal pocket to the human chest wall enhances the relevance of this model for assessing drug release kinetics. Each animal underwent anesthesia using a standard protocol, and a small incision was made in the dorsal surface. Blunt dissection was used to create a pocket to accept the implant. An antibiotic bioenvelope containing a dummy CIED (6.0 cm × 5.5 cm) was then inserted into the subcutaneous pocket and the incision was closed.

To monitor drug elution, device samples for pharmacokinetic analysis were collected at 2 h and 1, 3, 5, 7, and 14 days post-implantation. There were four animals for each time point. Animals were euthanized at predetermined intervals, and the devices were explanted and stored at −80 °C until analysis. Residual rifampin and minocycline in the explanted antibiotic bioenvelope were extracted by submerging in DMSO and agitated for 3 h at 25 °C. The resulting supernatant was filtered using a 0.45 µm PTFE filter and analyzed for each antibiotic using a validated high-performance liquid chromatography with ultraviolet detection (HPLC-UV, Shimadzu LC-2010HT HPLC System. Shimadzu Scientific Instruments, Columbia, MD, USA) method.

### 4.2. In Vitro Elution of Antibiotics

The in vitro elution procedure was conducted at 37 °C using phosphate-buffered saline (PBS) supplemented with sodium citrate to prevent sample degradation. To simulate the physiologic conditions of a subcutaneous environment and account for protein binding, 10% bovine serum albumin (BSA) was added to the PBS. Test samples were placed in amber-colored containers to minimize light exposure and degradation. Samples were subjected to agitation in sink conditions based on the solubilities of rifampin and minocycline. The elution process lasted 24 h, with elution samples collected at predefined intervals of 0.5, 1, 2, 4, 8, and 24 h. Each collected elution sample was analyzed using HPLC-UV to quantify the concentrations of rifampin and minocycline. The entire device and a sample of the bioenvelope containing only ECM without any disc material were also evaluated for drug content by extracting using the same method described for explanted samples from the in vivo PK study. Additionally, the binding of rifampin and minocycline to the matrix at equilibrium was assessed by analyzing the drug content of the envelope after 7 days at 37 °C under the same physiologic conditions. For this analysis, a 2.5 cm × 2.5 cm sample of the envelope matrix was extracted and tested.

### 4.3. HPLC-UV Quantification

HPLC analysis was conducted using a Shimadzu HPLC system equipped with an auto-sampler, column temperature controller, and UV detector. A Luna C18(2) column (250 × 4.6 mm, 5 µm particle size) was used for separation. The mobile phase consisted of 5 mM EDTA in water at pH 7.2 (mobile phase A) and 20% 3 mM EDTA in water with 80% methanol at pH 7.2 (mobile phase B). A flow rate of 1.5 mL/min was maintained, with an injection volume of 20 µL, and detection was carried out at a wavelength of 270 nm.

Stock solutions of minocycline and rifampin were prepared in DMSO at a concentration of 1 mg/mL. Working standards were prepared by serial dilution with the appropriate diluent to achieve concentrations ranging from 2 µg/mL to 160 µg/mL. Calibration curves were generated by plotting peak area versus concentration, achieving an R^2^ value of ≥0.99.

### 4.4. Determination of In Vitro–In Vivo Correlation

In vitro–in vivo correlation (IVIVC) was established by combining results of an in vivo study using a rabbit model with an in vitro elution test. The in vivo study was conducted over a duration of 2 weeks, the maximum period examined, to assess whether the desired antibacterial activity was sustained over this extended timeframe. The in vitro component simulated the antibiotic release profile over time, analogous to in vivo conditions. This correlation guided the development of the preconditioning procedure for the AATCC testing.

The IVIVC process involved correlating the levels of antibiotics implanted in the rabbit model with the measured residual drug quantities. After 14 days post-implantation, the residual drug amount was matched with a corresponding duration of in vitro elution (IVE). This correlation enabled the simulation of antibiotic levels in the antibiotic bioenvelope after 14 days in vivo. The in vivo and in vitro data were plotted similarly to a Levy plot to relate the extent of in vitro elution to the residual drug amount in vivo, and a best-fit line was used to establish this relationship. For the AATCC testing, the preconditioning duration was determined to be 24 h, corresponding to the antibiotic levels observed approximately 2 weeks post-implantation in vivo. This extended preconditioning aimed to demonstrate the devices’ efficacy in reducing bacterial activity after 2 weeks of implantation in vivo.

### 4.5. Preconditioning

Preconditioning involved the controlled elution of rifampin and minocycline until antibiotic levels corresponded to levels expected at the desired in vivo time point. Based on the IVIVC, devices were preconditioned for 24 h. The media contained PBS with 10% BSA to simulate the subcutaneous environment and account for potential protein binding effects in vivo, following the IVE testing methods.

### 4.6. Modified AATCC Test Method 100 Testing

The modified AATCC Test Method 100 was used to quantitatively assess antibacterial activity across seven bacterial strains relevant to CIED-related infections (Table 2): the Gram-positive organisms *S. aureus*, Methicillin-resistant *S. aureus* (MRSA), *Staphylococcus epidermidis*, and *Staphylococcus lugdunensis*; and the Gram-negative organisms *Acinetobacter baumannii*, *Stenotrophomonas maltophilia*, and *Escherichia coli* [41,42,43,44,45,46,47]. The selection of bacterial strains was based on their clinical relevance to CIED-related infections to ensure a comprehensive assessment of antibacterial effectiveness.

Bacterial cultures were incubated in tryptic soy broth (7 mL) for 24 h at 37 °C to achieve the inoculum density for testing. Three envelopes were assigned to each time point and organism, with three control envelopes at t = 0 h to assess the efficiency of extraction. The controls consisted of the same bioenvelopes that contained polymer discs, but without antibiotics. Bacterial growth was confirmed at t = 24 h using control samples.

After removing the drug discs, the bioenvelopes underwent a 2-stage neutralization step, followed by serial dilution and drop plating for quantitation. Dey-Engley neutralizing broth was used to neutralize the antimicrobial activity, ensuring accurate quantification of viable bacteria allowing for differentiation between true bacterial reduction and continued suppression due to residual antibiotic activity. The efficacy of this neutralization step was verified before testing, confirming the effective neutralization of any residual antimicrobial activity from the bioenvelopes.

Bacterial inoculation was conducted using 80 µL of the suspension with a target concentration of 1 × 10^6^ CFU/mL in tryptic soy broth, with control envelopes processed immediately and test envelopes incubated for 24 h before processing. The inoculation was performed peripheral to the discs. After incubation, the drug-eluting discs were removed, placed in the neutralization buffer, and samples were sonicated and plated for CFU counts. This process enabled the assessment of bacterial growth on the envelopes, allowing for a comparison between growth on coated envelopes and extraction from the controls. Log reductions in bacterial populations were calculated to evaluate the effectiveness of the antibiotic bioenvelope in preventing bacterial colonization.

To evaluate device performance over time, aged samples were tested at 3, 6, 9, and 12 months. Samples were subjected to real-time aging at 25 °C in their final packaging configuration. At each time point, samples were processed as described above and subjected to the modified AATCC-100 testing.

## 5. Conclusions

The results of this study support the use of antibiotic-eluting bioenvelopes as an effective strategy for preventing bacterial infections in CIED implants. Moreover, adaptation of the AATCC-100 test and the use of in vivo–in vitro correlation provide new tools for the evaluation of the antibiotic activity of implantable biomaterials.

## Figures and Tables

**Table 1:** Assay results for antibiotic bound to the bioenvelope.

	Disc		Matrix	
	Minocycline	Rifampin	Minocycline	Rifampin
Device 1	7.0%	14.7%	9.6%	13.4%
Device 2	6.1%	12.7%	9.8%	11.3%
Device 3	6.3%	13.9%	9.4%	10.8%
Mean	6.5%	13.8%	9.6%	11.8%

Note: Percentages are relative to nominal drug content.

**Table 2:** Microbial strains included in the AATCC study and their clinical relevance [41,42,43,44,46,47,48].

Type	Organism	Clinical Relevance	References
Gram-positive	*Staphylococcus aureus* ATCC 29213	Common cause of surgical site infections, including CIED implants in 30–35% of cases	Sohail 2007 [41] Hussein 2016 [42] Tarakji 2010 [43] Pichlmaier 2011 [44]
	Methicillin-resistant *S. aureus* (MRSA) ATCC 335912	Resistant variant of *S. aureus*, a significant concern in healthcare settings due to antibiotic resistance	Sohail 2007 [41]
	*Staphylococcus epidermidis* ATCC 35984	Causative agent in approximately one-third of CIED infections	Sohail 2007 [41] Hussein 2016 [42] Tarakji 2010 [43]
	*Staphylococcus lugdunensis* ATCC 49576	Coagulase-negative staphylococcus associated with skin and soft tissue infections	Sohail 2007 [41] Tarakji 2010 [43] Pichlmaier 2011 [44]
Gram-negative	*Acinetobacter baumannii* ATCC 19606	Opportunistic pathogen known for its ability to cause healthcare-associated infections; notable for its resilience and multidrug-resistant nature	Sohail 2007 [41] Antunes 2014 [46]
	*Stenotrophomonas maltophilia* ATCC 17666	Environmental bacterium with ability to adhere to and colonize medical device surfaces; associated with bloodstream infections and pneumonia	Sohail 2007 [41] Looney 2009 [47]
	*Escherichia coli* ATCC 25922	Pathogen capable of causing infections in various body sites, including the bloodstream and subcutaneous tissues	Chesdachai 2022 [48]

## Data Availability

The data presented in this study are available on request from the corresponding author.

## References

[1] Poole J.E., Gleva M.J., Mela T., Chung M.K., Uslan D.Z., Borge R., Gottipaty V., Shinn T., Dan D., Feldman L.A. (2010). Complication rates associated with pacemaker or implantable cardioverter-defibrillator generator replacements and upgrade procedures: Results from the REPLACE registry. Circulation.

[2] Han H.C., Hawkins N.M., Pearman C.M., Birnie D.H., Krahn A.D. (2021). Epidemiology of cardiac implantable electronic device infections: Incidence and risk factors. Europace.

[3] Sohail M.R., Eby E.L., Ryan M.P., Gunnarsson C., Wright L.A., Greenspon A.J. (2016). Incidence, Treatment Intensity, and Incremental Annual Expenditures for Patients Experiencing a Cardiac Implantable Electronic Device Infection: Evidence From a Large US Payer Database 1-Year Post Implantation. Circ. Arrhythm. Electrophysiol..

[4] Deering T.F., Chang C., Snyder C., Natarajan S.K., Matheny R. (2017). Enhanced Antimicrobial Effects of Decellularized Extracellular Matrix (CorMatrix) with Added Vancomycin and Gentamicin for Device Implant Protection. Pacing Clin. Electrophysiol..

[5] Sohail M.R., Esquer Garrigos Z., Elayi C.S., Xiang K., Catanzaro J.N. (2020). Preclinical evaluation of efficacy and pharmacokinetics of gentamicin containing extracellular-matrix envelope. Pacing Clin. Electrophysiol..

[6] Shiels S.M., Tennent D.J., Lofgren A.L., Wenke J.C. (2018). Topical rifampin powder for orthopaedic trauma part II: Topical rifampin allows for spontaneous bone healing in sterile and contaminated wounds. J. Orthop. Res..

[7] Tarakji K.G., Mittal S., Kennergren C., Corey R., Poole J.E., Schloss E., Gallastegui J., Pickett R.A., Evonich R., Philippon F. (2019). Antibacterial Envelope to Prevent Cardiac Implantable Device Infection. N. Engl. J. Med..

[8] Gomes F., Teixeira P., Ceri H., Oliveira R. (2012). Evaluation of antimicrobial activity of certain combinations of antibiotics against in vitro Staphylococcus epidermidis biofilms. Indian. J. Med. Res..

[9] Bowker K.E., Noel A.R., Macgowan A.P. (2008). Pharmacodynamics of minocycline against Staphylococcus aureus in an in vitro pharmacokinetic model. Antimicrob. Agents Chemother..

[10] Xiang K., Catanzaro J.N., Elayi C., Esquer Garrigos Z., Sohail M.R. (2021). Antibiotic-Eluting Envelopes to Prevent Cardiac-Implantable Electronic Device Infection: Past, Present, and Future. Cureus.

[11] Badylak S.F., Freytes D.O., Gilbert T.W. (2015). Reprint of: Extracellular matrix as a biological scaffold material: Structure and function. Acta Biomater..

[12] Brown B.N., Badylak S.F. (2014). Extracellular matrix as an inductive scaffold for functional tissue reconstruction. Transl. Res..

[13] American Association of Textile Chemists and Colorists TM 100 Test Method for Antibacterial Finishes on Textile Materials: Assessment 2019. https://members.aatcc.org/store/tm100/513/.

[14] Lee S.H., Glover T., Lavey N., Fu X., Donohue M., Karunasena E. (2024). Modified in-vitro AATCC-100 procedure to measure viable bacteria from wound dressings. PLoS ONE.

[15] Mellinghoff S.C., Bruns C., Albertsmeier M., Ankert J., Bernard L., Budin S., Bataille C., Classen A.Y., Cornely F.B., Couvé-Deacon E. (2023). Staphylococcus aureus surgical site infection rates in 5 European countries. Antimicrob. Resist. Infect. Control.

[16] Kalot M.A., Bahuva R., Pandey R., Farooq W., Mir A., Khan A., Kerling D., Aftab H., Kovacs A., Gupta S. (2023). Risk factors associated with higher mortality in patients with cardiac implantable electronic device infection. J. Cardiovasc. Electrophysiol..

[17] Wisplinghoff H., Bischoff T., Tallent S.M., Seifert H., Wenzel R.P., Edmond M.B. (2004). Nosocomial bloodstream infections in US hospitals: Analysis of 24,179 cases from a prospective nationwide surveillance study. Clin. Infect. Dis..

[18] Baddour L.M., Epstein A.E., Erickson C.C., Knight B.P., Levison M.E., Lockhart P.B., Masoudi F.A., Okum E.J., Wilson W.R., Beerman L.B. (2010). Update on cardiovascular implantable electronic device infections and their management: A scientific statement from the American Heart Association. Circulation.

[19] Kumar A., Doshi R., Shariff M. (2020). Role of antibiotic envelopes in preventing cardiac implantable electronic device infection: A meta-analysis of 14 859 procedures. J. Arrhythm..

[20] Ullah W., Nadeem N., Haq S., Thelmo F.L., Abdullah H.M., Haas D.C. (2020). Efficacy of antibacterial envelope in prevention of cardiovascular implantable electronic device infections in high-risk patients: A systematic review and meta-analysis. Int. J. Cardiol..

[21] Koerber S.M., Turagam M.K., Winterfield J., Gautam S., Gold M.R. (2018). Use of antibiotic envelopes to prevent cardiac implantable electronic device infections: A meta-analysis. J. Cardiovasc. Electrophysiol..

[22] Krahn A.D., Longtin Y., Philippon F., Birnie D.H., Manlucu J., Angaran P., Rinne C., Coutu B., Low R.A., Essebag V. (2018). Prevention of Arrhythmia Device Infection Trial: The PADIT Trial. J. Am. Coll. Cardiol..

[23] Rosenblatt J., Vargas-Cruz N., Reitzel R.A., Raad I.I. (2019). Assessment of the Potential for Inducing Resistance in Multidrug-Resistant Organisms from Exposure to Minocycline, Rifampin, and Chlorhexidine Used To Treat Intravascular Devices. Antimicrob. Agents Chemother..

[24] Jørgensen N., Skovdal S., Meyer R., Dagnæs-Hansen F., Fuursted K., Petersen E. (2016). Rifampicin-containing combinations are superior to combinations of vancomycin, linezolid and daptomycin against Staphylococcus aureus biofilm infection in vivo and in vitro. Pathog. Dis..

[25] Cavallo J.A., Greco S.C., Liu J., Frisella M.M., Deeken C.R., Matthews B.D. (2015). Remodeling characteristics and biomechanical properties of a crosslinked versus a non-crosslinked porcine dermis scaffolds in a porcine model of ventral hernia repair. Hernia.

[26] Reing J.E., Zhang L., Myers-Irvin J., Cordero K.E., Freytes D.O., Heber-Katz E., Bedelbaeva K., McIntosh D., Dewilde A., Braunhut S.J. (2009). Degradation products of extracellular matrix affect cell migration and proliferation. Tissue Eng. Part. A.

[27] Medberry C.J., Tottey S., Jiang H., Johnson S.A., Badylak S.F. (2012). Resistance to infection of five different materials in a rat body wall model. J. Surg. Res..

[28] Milburn M.L., Holton L.H., Chung T.L., Li E.N., Bochicchio G.V., Goldberg N.H., Silverman R.P. (2008). Acellular dermal matrix compared with synthetic implant material for repair of ventral hernia in the setting of peri-operative Staphylococcus aureus implant contamination: A rabbit model. Surg. Infect..

[29] Brennan E.P., Reing J., Chew D., Myers-Irvin J.M., Young E.J., Badylak S.F. (2006). Antibacterial activity within degradation products of biological scaffolds composed of extracellular matrix. Tissue Eng..

[30] Deegan D., Piasecki S.K., Riebman J. (2022). An Acellular Biologic Extracellular Matrix Envelope for Cardiovascular Implantable Electronic Devices: Preclinical Evaluation. J. Regen. Med..

[31] Wolf M.T., Carruthers C.A., Dearth C.L., Crapo P.M., Huber A., Burnsed O.A., Londono R., Johnson S.A., Daly K.A., Stahl E.C. (2014). Polypropylene surgical mesh coated with extracellular matrix mitigates the host foreign body response. J. Biomed. Mater. Res. A.

[32] Nayak H., Beaser A.D., Aziz Z.A. (2021). Patient Profiles in the Utilization of the CanGaroo(R) Envelope. Cureus.

[33] Holton L.H., Chung T., Silverman R.P., Haerian H., Goldberg N.H., Burrows W.M., Gobin A., Butler C.E. (2007). Comparison of acellular dermal matrix and synthetic mesh for lateral chest wall reconstruction in a rabbit model. Plast. Reconstr. Surg..

[34] Laschke M.W., Haufel J.M., Scheuer C., Menger M.D. (2009). Angiogenic and inflammatory host response to surgical meshes of different mesh architecture and polymer composition. J. Biomed. Mater. Res. B Appl. Biomater..

[35] Scislowska-Czarnecka A., Pamula E., Tlalka A., Kolaczkowska E. (2012). Effects of aliphatic polyesters on activation of the immune system: Studies on macrophages. J. Biomater. Sci. Polym. Ed..

[36] Lock A.M., Gao R., Naot D., Coleman B., Cornish J., Musson D.S. (2017). Induction of immune gene expression and inflammatory mediator release by commonly used surgical suture materials: An experimental in vitro study. Patient Saf. Surg..

[37] (2016). Biological Evaluation of Medical Devices—Part 6: Tests for Local Effects After Implantation.

[38] Hines D.J., Kaplan D.L. (2013). Poly(lactic-co-glycolic) acid-controlled-release systems: Experimental and modeling insights. Crit. Rev. Ther. Drug Carr. Syst..

[39] Makadia H.K., Siegel S.J. (2011). Poly Lactic-co-Glycolic Acid (PLGA) as Biodegradable Controlled Drug Delivery Carrier. Polymers.

[40] Hansen L.K., Brown M., Johnson D., Palme Ii D.F., Love C., Darouiche R. (2009). In vivo model of human pathogen infection and demonstration of efficacy by an antimicrobial pouch for pacing devices. Pacing Clin. Electrophysiol..

[41] Sohail M.R., Uslan D.Z., Khan A.H., Friedman P.A., Hayes D.L., Wilson W.R., Steckelberg J.M., Stoner S., Baddour L.M. (2007). Management and outcome of permanent pacemaker and implantable cardioverter-defibrillator infections. J. Am. Coll. Cardiol..

[42] Hussein A.A., Baghdy Y., Wazni O.M., Brunner M.P., Kabbach G., Shao M., Gordon S., Saliba W.I., Wilkoff B.L., Tarakji K.G. (2016). Microbiology of Cardiac Implantable Electronic Device Infections. JACC Clin. Electrophysiol..

[43] Tarakji K.G., Chan E.J., Cantillon D.J., Doonan A.L., Hu T., Schmitt S., Fraser T.G., Kim A., Gordon S.M., Wilkoff B.L. (2010). Cardiac implantable electronic device infections: Presentation, management, and patient outcomes. Heart Rhythm..

[44] Pichlmaier M., Knigina L., Kutschka I., Bara C., Oswald H., Klein G., Bisdas T., Haverich A. (2011). Complete removal as a routine treatment for any cardiovascular implantable electronic device-associated infection. J. Thorac. Cardiovasc. Surg..

[45] Garrigos Z.E., George M.P., Farid S., Abu Saleh O.M., Vijayvargiya P., Mahmood M., Friedman P.A., Steckelberg J.M., DeSimone D.C., Wilson W.R. (2018). Diagnostic evaluation and management of culture-negative cardiovascular implantable electronic device infections. Pacing Clin. Electrophysiol..

[46] Antunes L.C., Visca P., Towner K.J. (2014). Acinetobacter baumannii: Evolution of a global pathogen. Pathog. Dis..

[47] Looney W.J., Narita M., Muhlemann K. (2009). Stenotrophomonas maltophilia: An emerging opportunist human pathogen. Lancet Infect. Dis..

[48] Chesdachai S., Baddour L.M., Sohail M.R., Palraj B.R., Madhavan M., Tabaja H., Fida M., Lahr B.D., DeSimone D.C. (2022). Risk of cardiovascular implantable electronic device infection in patients presenting with gram-negative bacteremia. Open Forum. Infect. Dis..

